# Consistency of three different questionnaires for evaluating sexual function in healthy young women

**DOI:** 10.1186/s12905-018-0693-y

**Published:** 2018-12-20

**Authors:** Christiane Kelen Lucena da Costa, Maria Helena Constantino Spyrides, Maria Bernardete Cordeiro de Sousa

**Affiliations:** 1 Postgraduate Program in Health Sciences, Federal University of Rio Grande do Norte (UFRN), Natal, RN Brazil; 2 Department of Atmospheric and Climatic Changes, Federal University of Rio Grande do Norte (UFRN), Natal, RN Brazil; 3 Brain Institute Federal University of Rio Grande do Norte, Av. Nascimento de Castro 1255- Lagoa Nova, Natal, RN 59056-450 Brazil

**Keywords:** Female sexual function, Psychometric scales accuracy, Couple relationships, Primary diagnosis of dysfunction

## Abstract

**Background:**

Most studies on female sexual dysfunction are performed in population inventories and under specific clinical conditions. These approaches are performed using validated psychometric scales. Different scales to assess sexual function use different numbers of questions to characterize their domains. They also may or may not include domains of interaction between sexual partners. The objective of this study was to compare the precision between scales to be able to analyze their accuracy for better diagnosis of sexual dysfunction.

**Methods:**

Fifty (50) healthy young women were enrolled in this study. Three questionnaires (FSFI, SQ-F, and GRISS) were applied to assess sexual function (*n* = 44). The accuracy measured by the area under the ROC curve (AUC) for individual domains and to cross-validated pairwise comparison of the three analyzed instruments was used. Kruskall-Wallis test to analyze individual domains of the scales was also used.The *P*-value was established as 0.05.

**Results:**

The results showed that all domains and total FSFI and GRISS scores were significantly different between normal and dysfunctional women, but not for SQ-F domains. Indeed, AUC accuracy varied from excellent-good domain discrimination for FSFI and GRISS, but fair-poor for SQ-F. For the paired comparison between the three questionnaires a fair accuracy was detected. The specificity percentage was around 84% whereas that for sensibility was low, around 30%.

**Conclusions:**

The best agreement was between FSFI and SQ-F, probably being related to high similar shared questions when compared to GRISS. The agreement between SQ-F and GRISS was low possible due to low number of questions in SQ-F to characterize similar domains. This study evidenced high agreement between scales to sensitivity and low agreement for specificity, thereby conferring fair accuracy between them. Thus, the limited grade for discriminatory capacity (AUC) for sexual response should be considered when comparing results from these three different questionnaires and also when comparing with other different scales. In addition, despite the diversity of scales, the high reliability and fit for their desire domain suggest that the FSFI scale has good accuracy for the current clinical assessment of women’s sexual health.

**Clinical trial registration:**

NCT03241524. Retrospectively registered on 08/02/2017.

## Background

Most studies on female sexual dysfunction (FSD) are performed in population inventories and under specific clinical conditions such as postpartum, urinary incontinence, post-breast cancer surgery, obesity, and diabetes, among others [1–5]. These approaches are performed using validated psychometric scales, with the most frequently used being FSFI [6], GRISS [7] and, also, the SQ-F in the Brazilian population [8].

Female sexual dysfunction is a complex and multi-faceted disorder that has a wide spectrum of symptoms and severity and during their life cycle, women are frequently incapable or disinterested in participating in a sexual relationship. The causes are often multifactorial, requiring multidisciplinary assessment that addresses biological, psychological, sociocultural and relational aspects [9]. The concept of female sexual response has changed since Basson et al. [10] proposed the inclusion of an intimacy-based sex response cycle, in a nonlinear model that incorporates the value of emotional intimacy, sexual stimulus, and satisfaction with the relationship to replace the current linear model. In this context, in addition to a reproductive repertoire, sexuality comprises both physical and psychological feelings during part of a woman’s lifetime both for their own feeling as well as in relation to their partner [11]. In this perspective, scales which have included domains of interaction between sexual partners could have a higher chance to better diagnose sexual dysfunction.

According to the Fifth Edition of the Diagnostic and Statistical Manual [12] three disorders are cited: Female Orgasmic Disorder (FOD); Female Sexual Interest/Arousal Disorder (FSAD), and Genito-Pelvic Pain/Penetration Disorder (GPPPD). These disorders include four main domains for desire, arousal, orgasm, and pain (dyspareunia and vaginism), in which both physiological and psychological aspects of the female sexual experience influence the response [13].

Sexual dysfunctions are highly prevalent in the sexual disorders of both sexes according to Laumann, Paik & Rosen [14] who analyzed 1749 women and 1410 men, aged 18–59 years, living in the USA. The authors found that sexual dysfunction is more prevalent in women (43 to 34%), where the main complaints range from 27 to 32% for lack of interest in sex, 22–28% for inability to reach orgasm, 8–21% for pain during sex, and 17–27% for unpleasant sex.

For the Brazilian population, a study of 1219 women with a mean age of 35.6 + 12.31, observed that 49.0% exhibit at least one sexual dysfunction, 26.7% for desire, 23% dyspareunia and 21% for orgasm. In this study, educational level was inversely correlated with the risk of sexual desire, orgasm and pain dysfunctions during sexual intercourse [15]. In another cross-sectional study of 201 sexually active Brazilian women aged from 18 to 45 years, 90 from a public health service and 111 from private services, dysfunction occurred in 30.2% without a significant difference in the prevalence of sexual dysfunction or in sexual domain scores between income or educational status [16]. A prevalence of at least one type of sexual dysfunction in 36% of a sample of 100 women aged between 20 and 39 years old was also found [17]. Orgasm dysfunction occurred in 18% of the participants, and dyspareunia was reported by 13% of the women during the month prior to the study. Sexual appetite dysfunction occurred in 11% of the women, 8% had excitation dysfunction and only 1% reported vaginismus.

Even though there is high prevalence of sexual dysfunction in women, both physicians and patients are frequently hesitant to discuss this topic [18]. Moreover, according to reported sexual difficulties collected in the Global Study of Sexual Attitudes and Behaviors study 27,500 people (men and women aged 40–80 years) only around 19% (18.0% of men and 18.8% of women) sought medical help [19]. Sexual activity, sexual dysfunctions and related help-seeking behavior were investigated in adults (aged 40–80 years) using a telephone survey (5998 individuals; 2992 men and 3006 women) in 5 countries (United States, Canada, the United Kingdom, Australia, and New Zealand). Seventy-two percent (72%) of the respondents positive for dysfunction had no action in regards to it, and supposedly had a lack of perception or lack of discomfort about the problem. In addition, they did not know they needed to seek a doctor in such cases. Another cited cause was embarrassment (23%) [20]. Thus, this reflects lacking a favorable environment in both which men and women would feel not comfortable asking for information, and poses a challenge to physicians and other health professionals. This also indicates the importance of using questionnaires that have high precision resulting in accurate diagnoses and for effective monitoring treatments to counterbalance this gap [21, 22].

Sexual function has been evaluated by different surveys, but they may or not include domains of interaction with partners [21]. They also show a large variation in number of items that characterize each domain and they consequently vary in terms of psychometric properties. For example, FSFI is one of the most used questionnaires, but no domain related to the couple’s relationship is present. [6] On the other hand two others frequently used scales are the GRISS and SF-Q, with the latter being the most for the Brazilian population, covering issues where the quality of the couple’s relationships are addressed [7, 8]. Thus, the objective of this study was to compare the precision between scales which only quantify properties of female-specific sexual dysfunction and those which also score for quality of the couples’ relationship for diagnosing sexual dysfunction therefore directing the improvement of primary diagnosis of sexual disorders.

## Methods

### Study design and participants

This study is a quantitative descriptive applied research and used convenience sampling. The sample was composed of 60 female undergraduate students attending a private University in João Pessoa, northeastern Brazil, but only 50 agreed to participate in the study and also met the inclusion criteria. The inclusion criteria were: age between 19 and 35 years, heterosexual, active sex life, living in a stable relationship for at least 6 months no pregnancy or parturition in the last 6 months, clinically healthy and agreeing with the terms for participating in the study. Six women completed only 2 questionnaires and the final sample size for those women who filled out the three questionnaires was *n* = 44. The women were evaluated at the Physical Therapy Clinic and Laboratory of the Faculty where the study was conducted.

### Assessment of female sexual response

Three questionnaires that assess sexual function were used to compare the accuracy in detecting both functional and dysfunctional sexual evaluation. The FSFI was selected because it is the most widely used instrument [20]. SQ-F is an instrument validated for the target population of this study, and GRISS (similar to SQ-F) includes an assessment of interactions with the sexual partner as one of its items. The three questionnaires were completed by the participants and all were translated and validated for Portuguese, as follows: (1) Female Sexual Function Index (FSFI), based on Rosen et al. [6]; (2) Female Sexual Quotient (SQ-F) validated for the Brazilian population [8] and (3) Sexual Satisfaction Inventory, female version (GRISS) based on Rust & Golombok [7] and Pacagnella et al. [23] validated and adapted for the Brazilian population [24].

#### Female sexual function index (FSFI) questionnaire

This questionnaire was proposed by Rosen et al. [6] for the diagnosis of female sexual function. The instrument was validated and adapted to Portuguese and contains 19 questions with multiple-choice responses that assess sexual function in the last four weeks, associated with six domains and possible types of disorders: (a) desire, (b) arousal, (c) lubrication, (d) orgasm, (e) satisfaction with sexual life, and (f) pain during or after intercourse. Participants completed the instrument by choosing the option that best described their situation. Each question was associated with a value corresponding to the degree of gratification of the participant. A score of 0 indicates no sexual activity in the last four weeks, and the others are numbered 1 to 5 on an incremental scale. For the pain domain, the range of values from 1 to 5 is inversed while the grades vary from 0 to 5 on questions 3 to 14 and 17 to19, and from 1 to 5, and for questions 1,2,15 and 16. The overall score is the sum of each domain multiplied by its corresponding factor and ranges from 2 to 36. Total scores smaller than 26 indicate one or more dysfunction in the specific domains.

#### SQ-F questionnaire (SQ-F)

SQ-F is an instrument developed and validated for Brazilian women by Abdo [8] and is composed of 10 questions that assess sexual function, addressing desire and interest in sex (questions 1, 2, 8), foreplay (question 3), sexual arousal and harmony with the partner (questions 4, 5), comfort in sexual intercourse (questions 6, 7) and orgasm and sexual satisfaction (questions 9, 10). The overall scores range from 0 to 100, in accordance with sexual performance, where: 82–100 = good to excellent; 62 |– 82 = regular to good; 42 |- 62 = unfavorable to regular; 22 |- 42 = bad to unfavorable; 0 |- 22 null to bad. A total score smaller than 62 indicates a poor sexual relationship.

#### Golombok-Rust inventory of sexual satisfaction (GRISS)

The GRISS questionnaire was developed to evaluate sexual dysfunction in heterosexual couples, discriminating between those with and without sexual difficulties. It is a 28-item questionnaire that produces a total GRISS score as well as two separate scales for both males and females. The scale has 8 items for females, including anorgasmia, vaginismus, non-communication, infrequency, female avoidance, female non-sensuality, female dissatisfaction, and anorgasmia [7]. The score ranges from 0 to 10, with values ​​greater than 5 indicating sexual dysfunction.

### Statistical analysis

To analyze the differences between each domain within each of the three scales, the Kruskall-Wallis nonparametric test was used to compare medians calculated in the group that presented the typical sexual response and, in the group, where the sexual response was dysfunctional. A *p*-value < 0.05 was established.

The diagnostic performance or accuracy of a test in discriminating disease cases from normal cases is evaluated using Receiver Operating Characteristic (ROC) curve analysis [25]. A receiver operating characteristics (ROC) graph is a technique for visualizing, organizing and selecting classifiers based on their performance. ROC analysis has been extended to visualize and analyze the behavior of diagnostic systems [26]. ROC curves can also be used to compare the diagnostic performance of two or more laboratory or diagnostic tests. The closer the ROC curve is to the upper left corner, the higher the overall accuracy of the test [26, 27].

The curve is created by plotting the true positive rate (TPR) against the false positive rate (FPR) at various threshold settings. The true-positive rate is also known as sensitivity, or recall in machine learning. The false-positive rate is also referred to as the fall-out and can be calculated as (1-specificity). Thus, the ROC curve is sensitivity as a function of fall-out.

Accuracy is measured by the area under the ROC curve (AUC) and is used in classification analysis to determine which of the models best predicts the classes. An area of 1 represents a perfect test and 0.5 a worthless test. A rough guide for classifying the accuracy of a diagnostic test is the traditional academic point system: 0.90–1 = excellent; 0.80–0.90 = good; 0.70–0.80 = fair; 0.60–0.70 = poor; 0.50–0.60 = fail.

The receiver operating characteristic (ROC) curve was used to test accuracy in predicting sexual function within domains and among the three instruments applied to evaluate female sexual response.

## Results

In regard to relationship status, 60% of the participants reported being single (*n* = 30), 30.0% married (*n* = 15) and five divorced (10.0%). Fifty-six percent (56%) identified themselves as white (*n* = 28), 28.0% brown and 16.0% black. In relation to life style, 35.4% (*n* = 17) of the sample were social drinkers, 100% (*n* = 48) non-smokers, 70.8% sedentary. There were 60.4% (*n* = 29) who did not use birth control pills and 20.8% (*n* = 10) had children.

Within the sample, 18.8% (*n* = 9) of the women exhibited sexual dysfunctions according to the FSFI. Analysis using the SQ-F detected 7 women (14.6%) with sexual dysfunction, whereas the GRISS Inventory also found that 18.2% (*n* = 8) of the women presented dysfunctions.

### Analysis by sexual response domains in the FSFI, SQ-F and GRISS questionnaires

For the three questionnaires, the total and individual means of scores by domains for functional and dysfunctional women are shown in Table 1. The Kruskall-Wallis test showed that FSFI differences between functional and dysfunctional women in relation to measured sexual variables (Desire, *p*-value < 0.05; Arousal, Lubrification, Satisfaction, Orgasm and Pain, p-value < 0.01) were significant. For SQ-F scale, significant differences between women with dysfunction and those without were not observed for any domain. Analysis for individual domains and total scores for GRISS showed significant differences in all (Infrequency, p-value < 0.05; No-Communication, Dissatisfaction, Avoidance, No-sensuality, Vaginism and Anorgasmia, *p*-value < 0.01).

**Table 1:** Scores for individual domains/variables and total values obtained using FSFI and SQ-F questionnaires for female sexual response, and scores obtained for sexual quality of life and sexual disfunction using the GRISS inventory

Questionnaires	Total	No dysfuncion	Dysfunction
FSFI			
Desire^a^	4.7 ± 0.1	4.8 ± 0.2	4.1 ± 0.3
Arousal^b^	4.8 ± 0.1	5.1 ± 0.1	3.4 ± 0.5
Lubrication^b^	4.7 ± 0.2	4.9 ± 0.1	3.7 ± 0.5
Satisfaction^b^	5.3 ± 0.1	5.5 ± 0.1	4.6 ± 0.2
Orgasm^b^	4.7 ± 0.2	4.9 ± 0.2	3.6 ± 0.5
Pain^b^	4.6 ± 0.2	5.1 ± 0.1	2.3 ± 0.5
Total^b^	**28.9 ± 0.7**	**30.5 ± 0.4**	**21.7 ± 1.9**
SQ-F	Total	No dysfuncion	Dysfunction
Sexual Interest	11.3 ± 0.4	11.4 ± 0.4	10.4 ± 0.8
Preliminaries	4.8 ± 0.1	4.8 ± 0.1	4.9 ± 0.1
Arousal	8.9 ± 0.2	9.0 ± 0.2	8.3 ± 1.2
Satisfaction	8.3 ± 0.2	8.4 ± 0.2	7.3 ± 0.8
Comfort	4.4 ± 0.1	4.5 ± 0.1	4.1 ± 0.4
Total	**80.6 ± 2.2**	**81.5 ± 2.3**	**84.0 ± 2.2**
GRISS	Total	No dysfuncion	Dysfunction
Infrequency^a^	4.1 ± 0.3	3.8 ± 0.3	5.3 ± 0.5
No-communication^b^	2.9 ± 0.4	2.4 ± 0.4	5.3 ± 0.8
Dissatisfaction^b^	5.2 ± 0.5	4.3 ± 0.5	9.1 ± 0.6
Avoidance^b^	3.4 ± 0.5	2.5 ± 0.4	7.4 ± 0.6
Non-sensuality^b^	2.4 ± 0.4	1.4 ± 0.3	6.6 ± 1.1
Vaginismus^b^	5.5 ± 0.6	4.7 ± 0.6	9.1 ± 1.6
Anorgasmia^b^	6.0 ± 0.6	4.9 ± 0.5	10.5 ± 0.8
Total^b^	**3.2 ± 0.3**	**2.5 ± 0.3**	**6.4 ± 0.2**

^a^significant difference at 5%; ^b^significant difference at 1%; The total values ​​for the domains of each questionnaire are in bold

With respect to the use of the three questionnaires to evaluate participants’ sexual dysfunction, most domains of each instrument showed good discriminatory capability using ROC analysis (Table 2). AUC values varied from excellent (0.90|-1.00) (FSFI: Arousal and Pain; and GRISS: Non-sexuality, Dissatisfaction) to fail (0.50|-0.60) (SQ-F: Preliminaries and Comfort). A good AUC rating (0.80|-0.90) was observed for FSFI (Lubrification, Satisfaction and Orgasm), and GRISS (Non-communication, Vaginismus, Anorgasmia). A fair AUC grade was detected for FSFI (Desire), GRISS (Infrequency) and SQ-F (Sexual interest, Satisfaction) and a poor AUC grade for SQ-F (Arousal).

**Table 2:** Sensitivity, Specificity and percentage of the area under the curve obtained by ROC analysis related to each domain for the 3 instruments used to assess sexual response

Domains	Sensibility	Specificity	AUC
(%)	(%)	Probability	
FSFI			
Desire	62.5	69.4	0.705
Lubrication	75.0	91.7	0.833
Arousal	100.0	83.3	0.967
Pain	100.0	75.0	0.936
Satisfaction	100.0	66.7	0.865
Orgasm	75.0	83.3	0.847
QS-F			
Sexual interest	100.0	57.5	0.762
Preliminaries	25.0	82.5	0.534
Arousal	50.0	100.0	0.631
Comfort	50.0	87.5	0.519
Satisfaction	50.0	90.0	0.753
GRISS			
Infrequency	100.0	36.1	0.745
No-communication	62.5	86.1	0.814
Dissatisfaction	87.5	83.3	0.915
Avoidance	87.5	80.6	0.891
Non-sensuality	75.0	94.4	0.931
Vaginismus	75.0	86.1	0.806
Anorgasmia	87.5	80.6	0.814

**Fig. 1:**
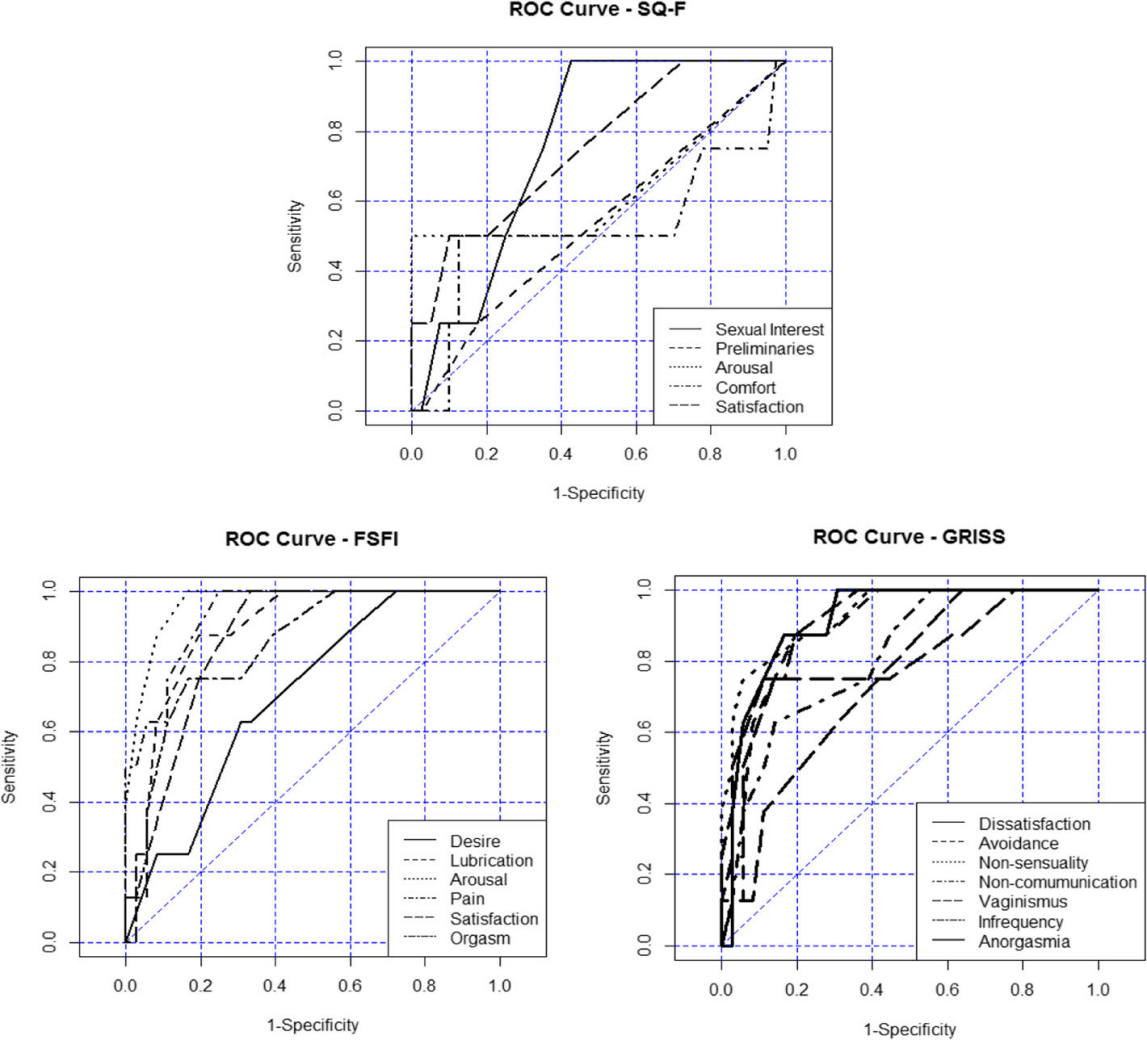
ROC curve Sensitivity and Specificity to predict the power of the three instruments (FSFI, SQ-F and GRISS Inventory - female scale), in relation to their female sexual function domains

The SQ-F domains had a lower discriminatory capacity to indicate sexual dysfunction compared to the GRISS and FSFI domains, meaning that the domains of the last two instruments more accurately discriminated the sexual dysfunction of women. Figure 1 shows the graphical representation of the domains for each questionnaire and indicates that the closer the point approaches the upper left corner of the graph, the greater its ability to correctly estimate women who have the presence or absence of sexual dysfunction.

### Analysis of the agreement between FSFI, SQ-F and GRISS questionnaires

**Fig. 2:**
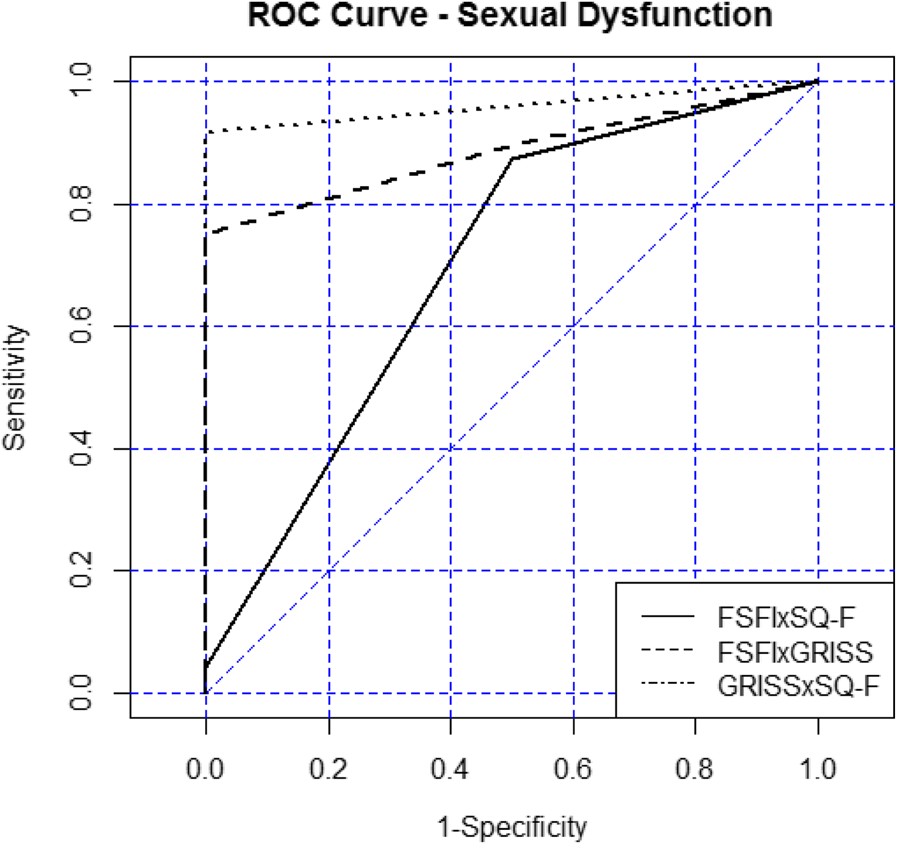
ROC curve sensitivity and specificity to predict the power of one instrument versus the other, regarding the FSFI, SQ-F and GRISS female scales

Classifying a high-frequency diagnosis of sexual dysfunction of one particular instrument with another instrument that did not diagnose (false positive) and classify as no dysfunction when another instrument diagnosed dysfunction (false negative), demonstrates incompatibilities in the results of these instruments. Thus, to achieve the best combination of sensitivity and specificity, the results were analyzed and can be observed by the ROC curve (Fig. 2). As already mentioned, the closer the point approaches the upper left corner of the graph, the greater the instrument’s ability to correctly estimate women’s sexual disorders.

The statistical values for comparison between questionnaires in terms of sensibility (truth-positive, i.e. typical sexual function) and specificity (truth-negative, i.e. sexual dysfunction) indicate the agreement between each other and are shown in Table 3. The agreement between FSFI and SQ-F was 84.6% in detecting of women with normal sexual function and 33.3% in those with dysfunction, with an accuracy of 0.778 and a discriminatory capacity of around .59. The agreement between FSFI and GRISS was 28.6% for the diagnosis of true sexual dysfunction and 82.9% for women without sexual dysfunction. According to the results, the precision between these instruments was 0.738 and AUC = 0.557. The agreement between the GRISS and SQ-F questionnaires varied in terms of classifying women with dysfunction (25.0% sensitivity) and no dysfunction (82.1% specificity). Thus, the agreement between them was acceptable and accurate to 0.767, but with a regular discriminatory ability (AUC = 0.535). Thus, all these results were classified above 0.70, indicating good concordance between instruments with the highest agreement in predicting typical sexual function as well as sexual dysfunction was between FSFI x SQ-F (Table 3).

**Table 3:** Percentage of agreement between questionnaires and accuracy for the comparison

Comparison	Specificity	Sensibility	Accuracy	Discriminatory capacity (AUC)
FSFI x SQ-F	84.6%	33.3%	0.778	0.590
FSFI x GRISS	82.9%	28.6%	0.738	0.557
GRISS x SQ-F	82.1%	25.0%	0.767	0.535

## Discussion

According to the results of this study, all three questionnaires detected sexual dysfunction or its absence. The results showed “excellent to good”’ domain discrimination for FSFI and GRISS, but “fair to poor” for SQ-F. However, agreement for paired-comparison between the three questionnaires showed a similar fair accuracy for the detected sexual response. These findings indicate that even those questionnaires that have an excellent to fair accuracy in most domains (FSFI and GRISS), and that which show “fair to poor” agreement in detected dysfunction (SQ-F) are fair in accordance. This might have occurred because some domains need to be reviewed in terms of their detection ability in these scales. For instance, the desire domain shows low reliability [21] on the FSFI questionnaire, as well as in the present study, where it showed a fair probability (low sensibility and specificity) when compared to its other domains which were excellent or good. A similar specificity grade (fair) for desire was also observed for SQ-F and was probably higher due to this similarity agreement between both questionnaires. Moreover, the proximity among the domains explored by them are higher than those of GRISS.

The domains explored by GRISS are more general and also included the feelings about the partners while FSFI has only specific female psychometric properties and does not explore a partnerships evaluation.

Although the prevalence of sexual dysfunction increases in middle-age and menopause, a significant percentage of sexual dysfunctions are also reported for young women. For instance, in using a sample of 201 sexually active Brazilian women aged 18 to 45 years, Prado, Mota & Lima [16] demonstrated that sexual dysfunction affected 30.2% of these individuals. Similarly, Ferreira, Souza & Amorim [17] found that 36% of women aged between 20 and 39 years exhibit at least one type of sexual dysfunction, 18% for orgasm and 13% for dyspareunia.

Around 18% of the sample of the present study showed sexual dysfunctions and the concordance between questionnaires was around 30%, but increases to 80% for characterizing typical sexual function. Therefore, the use of questionnaires to investigate sexual function in women should be re-evaluated and updated to improve their reliability for diagnoses if necessary.

In a study where the scales for diagnosing female sexual dysfunction were systematized, 27 available scales were listed, of which ten measure psychometric properties of specific domains of sexual dysfunction in women [20]. Of these, five presented reliability scores equal to or above 0.9 (Cronbach’s alpha), including FSFI. However, only the FSFI [6] contains items that contemplate the general domains to characterize the female sexual response included in the DSM-5 [12]. For example, one scale specifically addressed a cut-off point for the desire disorder domain in FSFI in order to achieve high sensitivity and specificity. Both indices increased from 75 and 84% to 92 and 89%, respectively. This scale (FSFI), as previously mentioned, has a moderate discriminative effect for this domain [28]. The other scales showed good scores for arousability, [29] impact of sexual dysfunction on women’s sexual life quality, [30] and to exclusively diagnose hypoactive sexual desire disorder (HSDD) [31] or HSDD due female distress [32]. From this evidence, application of the FSFI scale associated to a correction of the desire sexual domain is recommended [28].

The present study has limitations in estimating the sensitivity of inter-questionnaire agreement, likely due to the sample size and the fact that only young women were assessed. Also, data generalization is restricted since the sampling was performed by convenience. However, this study showed evidence of a need to improve the questionnaires that are presently being used to better characterize female sexual function Although more complete protocols have been proposed in relation to the main aspects of sexual function for constructing guidelines of the sexual history of men and women for health care professionals, [33] at the moment this protocol is not available in the public health service. Thus, the high reliability and adjustments introduced in the FSFI seem to provide a good instrument for its current use in the clinical diagnosis of the sexual health of women.

## Conclusions

A comparison of accuracy to detect typical sexual function and sexual dysfunction was investigated using three widely used questionnaires, namely the FSFI, GRISS and SQ-F. Precision grades from “excellent to good” were detected for most FSFI and GRISS individual domains, and “fair to poor” accuracy for SQ-F. Despite these results, the comparison between each 2 scales using the ROC curve to test the accuracy in predicting female sexual response diagnoses, were similar, being measured as “fair”. The sensitivity and specificity between FSFI x SQ-F were 84.6 and 33.3%, respectively, whereas between FSFI and GRISS were 82.9 and 28.6% and GRISS x SQ-F 82.1 and 25.0%. The number of questions per domains of the scales ranged from one to four, and it was found that lower scores for a domain for the FSFI were also low for a similar domain in the SQ-F. In addition, these two questionnaires have high similar questions when compared to GRISS. As SQ-F and GRISS contain items related to the sexual partnership a better agreement between them was expected. However, they were the lowest which might be due to the low number of questions on the SQ-F to characterize similar domains. This study evidenced high agreement between scales to truth-positive symptoms and low agreement for truth-negative symptoms thereby conferring fair accuracy between the studied scales. The limited grade for discriminatory capacity (AUC) between surveys for sexual response must be considered when comparing results from these three different questionnaires and also to compare with other different scales. In addition, despite the diversity of scales, the high reliability and fit for their desire domain suggest that the FSFI scale has good accuracy for the current clinical assessment of women’s sexual health.

## Abbreviations

FSFI: Female Sexual Function Index
SQ-F: Female Sexual Quotient
GRISS: Golombok-Rust Inventory of Sexual Satisfaction
ROC: Receiver-operating characteristic
